# The complete mitogenome of ‘Fujian’: an important *Saccharina japonica* cultivar of South China

**DOI:** 10.1080/23802359.2018.1473735

**Published:** 2018-05-15

**Authors:** Jing Zhang, Na Liu, Tao Liu

**Affiliations:** a Qilu University of Technology (Shandong Academy of Sciences), Jinan, Shandong Province, People’s Republic of China;; b College of Marine Life Sciences, Ocean University of China, Qingdao, Shandong Province, People’s Republic of China

**Keywords:** Cultivar ‘Fujian’, comparative analyses, genetic origin, mitogenome, phylogenetic relationship

## Abstract

In this work, complete mitogenome of *Saccharina japonica* cultivar ‘Fujian’ was reported. It had circular mapping organization with the length of 37,638 bp and encoded 66 genes including three rRNAs, 25 tRNAs, 35 known proteins, and three ORFs. Gene arrangement and component were conserved in those reported *Saccharina* species and cultivars. Only 33 nucleotide substitutions were found according to the total alignment of *S. japonica* and ‘Fujian’. The intergenic region of 19 nucleotides which is located between *rps*3 and *rps*9 in *S. japonica* mitogenome was not detected in ‘Fujian’. Phylogenetic analysis showed that ‘Fujian’ had a closer relationship with *S. japonica* and cultivar ‘Rongfu’ which strongly supported its genetic origin and phylogenetic relationship of Chinese *Saccharina* cultivars.

*Saccharina* (Laminariales, Phaeophyceae) is one of the most important seaweeds owing to its economic importance and global distribution (Kain 1979). In China, about 20 *Saccharina* cultivars have been bred (Li et al. 2007; Zhang et al. 2007, 2011b) and most of them were selected from *S*. *japonica*. ‘Fujian’ mainly cultured in South China was a *S. japonica* cultivar without any systematic selection. Here, we characterized the complete mitogenome of ‘Fujian’, gave the comparison with that of *S. japonica* (accession number: AP011493) and construct phylogenetic analysis to provide new molecular data for genetics study.

‘Fujian’ specimen (specimen number: 201005125) was collected from Putian, Fujian, China (25°43′N, 119°00′E) and stored at –80 °C for DNAs isolation. The experimental procedures and data processing followed Zhang et al. (2011a).

The ‘Fujian’ mitogenome was a circular molecule of 37,638 bp (GenBank accession number KX073815). The overall AT content was 64.66% exhibiting a high AT richness. Cumulative GC-skew and AT-skew analysis reflected a slight bias towards G and T on H-strand. The protein-encoding regions were 29,007 bp, the spacer size was 2426 bp and the overlap was 95 bp. One obvious gene cluster (*rps*8–*rpl*6–*rps*2–*rps*4) conserving in the reported *Saccharina* mitogenomes was also found here. The mitogenome encoded 66 genes, including three rRNAs (23S, 16S, and 5S), 25 tRNAs, 35 protein-encoding genes, and three ORFs. With the exception of *rpl*2, *rpl*16, *rps*3, *rps*19, *tat*C, and ORF130, 60 genes were encoded on H-strand. The universal genetic codes were used. All protein-encoding genes started with ATG codon. Approximately, 68.42% terminated with TAA codon, higher than that for TAG (21.05%) and TGA (10.53%). Moreover, all tRNA sequences could form standard clover-leaf secondary structures. Gene arrangement and component were identical with those reported *Saccharina* mitogenomes (Yotsukura et al. 2010; Zhang et al. 2013, 2017).

Compared to the mitogenome of *S. japonica*, 33 nucleotide substitutions were found in ‘Fujian’. One intergenic region of 19 nucleotides, which was located between *rps*3 and *rps*9 in *S. japonica*, lacked from ‘Fujian’ mitogenome. This resulted in the mitogenome size of ‘Fujian’ being 19 bp shorter than that of *S. japonica*.

wBayesian analysis based on the whole mitogenome sequences shared by 17 *Saccharina* and *Laminaria* algae was utilized to reconstruct the phylogeny (Figure 1). *Ectocarpus siliculosus* was used as outgroup. All algae were divided into two clades: *Saccharina* (including all Chinese cultivars) and *Laminaria*. Phylogenetic analyses showed that ‘Fujian’ firstly groups with *S. japonica* and ‘Rongfu’ which supported its genetic origin and relationship of Chinese cultivars.

**Figure 1.**
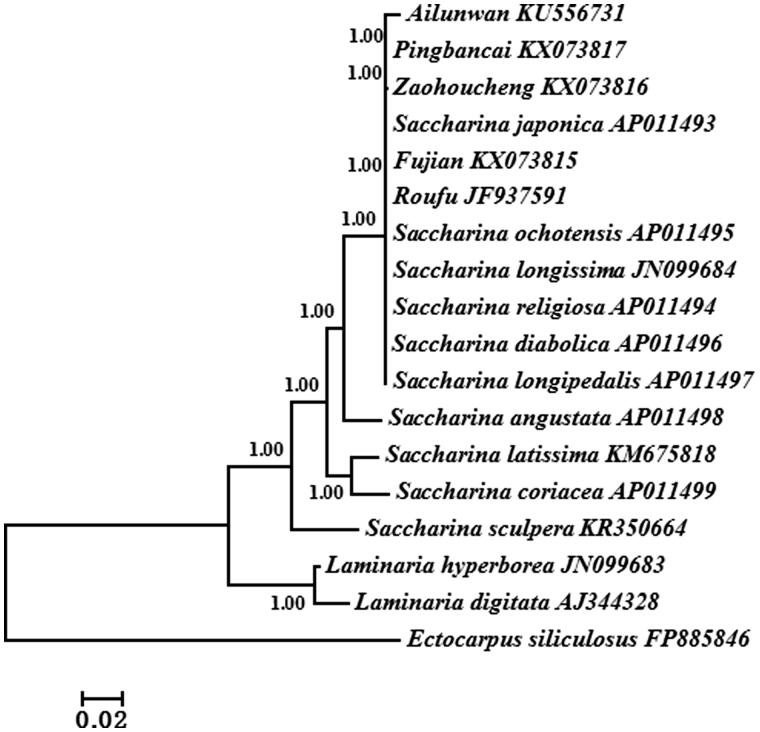
Phylogenetic tree constructed based on combined 35 mtDNA protein-encoding genes using Bayesian analysis.
